# C9ORF72 knockdown triggers FTD-like symptoms and cell pathology in mice

**DOI:** 10.3389/fncel.2023.1155929

**Published:** 2023-04-17

**Authors:** Maria-Belen Lopez-Herdoiza, Stephanie Bauché, Baptiste Wilmet, Caroline Le Duigou, Delphine Roussel, Magali Frah, Jonas Béal, Gabin Devely, Susana Boluda, Petra Frick, Delphine Bouteiller, Sébastien Dussaud, Pierre Guillabert, Carine Dalle, Magali Dumont, Agnes Camuzat, Dario Saracino, Mathieu Barbier, Gaelle Bruneteau, Phillippe Ravassard, Manuela Neumann, Sophie Nicole, Isabelle Le Ber, Alexis Brice, Morwena Latouche

**Affiliations:** ^1^Institut du Cerveau–Paris Brain Institute–ICM, Inserm, CNRS, Paris, France; ^2^German Center for Neurodegenerative Diseases (DZNE), Tübingen, Germany; ^3^Department of Neuropathology, Tübingen University Hospital, Tübingen, Germany; ^4^EPHE, Neurogenetics Team, PSL Research University, Paris, France

**Keywords:** TDP-43, C9ORF72, FTD (frontotemporal dementia), ALS (amyotrophic lateral sclerosis), autophagy/lysosomal pathway

## Abstract

The GGGGCC intronic repeat expansion within *C9ORF72* is the most common genetic cause of ALS and FTD. This mutation results in toxic gain of function through accumulation of expanded RNA foci and aggregation of abnormally translated dipeptide repeat proteins, as well as loss of function due to impaired transcription of *C9ORF72*. A number of *in vivo* and *in vitro* models of gain and loss of function effects have suggested that both mechanisms synergize to cause the disease. However, the contribution of the loss of function mechanism remains poorly understood. We have generated C9ORF72 knockdown mice to mimic C9-FTD/ALS patients haploinsufficiency and investigate the role of this loss of function in the pathogenesis. We found that decreasing C9ORF72 leads to anomalies of the autophagy/lysosomal pathway, cytoplasmic accumulation of TDP-43 and decreased synaptic density in the cortex. Knockdown mice also developed FTD-like behavioral deficits and mild motor phenotypes at a later stage. These findings show that C9ORF72 partial loss of function contributes to the damaging events leading to C9-FTD/ALS.

## Introduction

A hexanucleotide repeat expansion (HRE) located in the 5′ UTR region of *C9ORF72* gene is the most common genetic cause of familial frontotemporal dementia (FTD) and amyotrophic lateral sclerosis (ALS) (DeJesus-Hernandez et al., 2011; Renton et al., 2011; Gijselinck et al., 2012). These two fatal neurodegenerative diseases have been known to occur within the same families or patients, and the discovery of C9-FTD/ALS has strongly emphasized the existence of a clinical, genetic, pathological, and mechanistic continuum between FTD and ALS (Vance, 2006; Burrell et al., 2016).

The expanded GGGGCC impairs normal transcription of *C9ORF72* leading to reduced C9ORF72 mRNA and protein in the frontal cortex and spinal cord of patients (DeJesus-Hernandez et al., 2011; Gijselinck et al., 2012; Belzil et al., 2013; van der Zee et al., 2013; Waite et al., 2014; Xiao et al., 2015; Sivadasan et al., 2016). However, transcription of the non-coding HRE and its antisense sequence leads to two concomitant gain of function mechanisms, in addition to the loss of function. Aggregation of the expanded sense and antisense RNA in foci sequesters RNA-binding proteins, impedes their normal function, and thus, eventually leads to cell death (Donnelly et al., 2013; Lee et al., 2013; Sareen et al., 2013; Loureiro et al., 2016). Translation of aggregating peptides produced by repeat-associated non-AUG (RAN) translation (Ash et al., 2013; Mori et al., 2013) into dipeptide repeat proteins (DPRs) can variably cause degeneration in cell culture, drosophila and mouse models (Ash et al., 2013; Mori et al., 2013; Zu et al., 2013; Mizielinska et al., 2014; Wen et al., 2014; Zhang et al., 2014, 2016; Freibaum et al., 2015; Tran et al., 2015; Freibaum and Taylor, 2017; Schludi et al., 2017; Hao et al., 2019; LaClair et al., 2020). Both the RNA foci and the DPRs are present in the brains of patients carrying the *C9ORF72* mutation (DeJesus-Hernandez et al., 2011; Gendron et al., 2015; Gomez-Deza et al., 2015; Mackenzie et al., 2015).

Despite conflicting results in the different models regarding the precise identity of the most toxic species among extended RNA or DPRs, it is now established that those gain of function entities can cause neurodegeneration (Balendra and Isaacs, 2018). Nevertheless, it remains unclear if the disease is in fact caused solely by the gain of function mechanisms. Etiological evidence remains elusive in patients where DPR pathology is hardly correlated with affected regions (Mackenzie et al., 2013; Mann et al., 2013; Gomez-Deza et al., 2015). Mammalian gain of function models have generally failed to fully reproduce the C9-FTD/ALS histopathological lesions and associated behavioral changes (Balendra and Isaacs, 2018). Bacterial artificial chromosome (BAC) transgenic mice mainly reproduce RNA foci formation and DPR inclusions, but only rare or unstable TDP-43 pathology, neurodegeneration or FTD/ALS phenotype (O’Rourke et al., 2015; Peters et al., 2015; Jiang et al., 2016; Liu et al., 2016; Mordes et al., 2020; Nguyen et al., 2020). In other models, both histopathology and some behavioral modifications related to FTD/ALS were observed but only when overexpressing the HRE (Chew et al., 2015; Herranz-Martin et al., 2017) or specific DPRs (Zhang et al., 2016; Schludi et al., 2017; Choi et al., 2019; Hao et al., 2019; LaClair et al., 2020).

Therefore, the loss of function mechanism that initially seemed an unlikely disease trigger has recently regained attention and is being further explored (Lutz, 2020). The precise function of C9ORF72 remains largely unknown, but is predicted to be a guanine nucleotide exchange factor (GEF) interacting with various Rab proteins and forming a complex with SMCR8 and WDR41 to regulate membrane trafficking and autophagy/lysosomal flux (Levine et al., 2013; Amick et al., 2016; Blokhuis et al., 2016; Sellier et al., 2016; Sullivan et al., 2016; Webster et al., 2016; Xiao et al., 2016; Yang et al., 2016). The first *in vivo* models of *C9orf72* loss of function in zebrafish and *Caenorhabditis elegans* resulted in locomotor phenotypes and motoneuron degeneration (Ciura et al., 2013; Therrien et al., 2013). On the other hand, knockout mice predominantly developed an inflammatory phenotype, sometimes associated with a shortened lifespan, but did not exhibit neurodegeneration or motor phenotypes (Koppers et al., 2015; Burberry et al., 2016; O’Rourke et al., 2016; Sudria-Lopez et al., 2016; Sullivan et al., 2016; Ugolino et al., 2016). Abnormal social recognition and mild motor deficits were nevertheless noticed in two studies (Atanasio et al., 2016; Jiang et al., 2016) and late learning and memory deficits were recently characterized in *C9orf72* null mice, associated with enhanced synaptic pruning (Lall et al., 2021). While it appears that C9ORF72 deficiency is not the sole or more potent trigger of neurodegeneration in C9-FTD/ALS, its exact contribution to the phenotype has not yet been fully understood. Very recently, it was clearly demonstrated that the loss or lowering of C9ORF72 is an essential contributor to the development of the disease, both in human neurons and in rodent models (Shi et al., 2018; Shao et al., 2019; Staats et al., 2019; Dong et al., 2020a,b; Zhu et al., 2020). However, the behavioral and neurological impact of lowering C9ORF72 expression to levels observed in patients, in the absence of gain of function toxicities, is still largely lacking. In particular, the possibility that decreasing C9ORF72 expression, instead of proceeding to a full excision, could be enough to trigger some late or subtle anomalies has not been fully explored, especially regarding FTD-like phenotypes. We have investigated this by generating a ubiquitous knockdown (KD) mouse model and performing extensive behavioral and histological characterization. We found that KD mice developed pathological signs of C9-FTD/ALS, behavioral deficits in social interaction and depression-like behavior, as well as a lessening of strength and neuromuscular junction abnormalities that appeared at an advanced age.

## Materials and methods

Please see the Supplementary Information for detailed procedures.

### C9ORF72 miR-RNAi generation

Generation of miR-RNAi anti-*C9orf72* (miR-*C9orf72*) mice was done using a lentiviral vector carrying a ubiquitous PGK promoter driving the expression of the 5′-TTGACATCCACATCAATGTGCGTTTTGGCCACTGACTGAC GCACATTGGTGGATGTCAA-3′ sequence targeting mouse C*9orf72* transcript variants 1, 2, and 3, coupled to the expression of EmGFP. It was injected in C57Bl6/N mice oocytes that were then implanted in pseudopregnant females as previously described (Dussaud et al., 2018). A similar vector carrying a random miR-RNAi sequence was used for scramble controls (miR-Scramble).

All animal experiments were approved by the institutional animal care and use committee CEEA –005 and in agreement with the European legislation No. 2010/63 UE and national authority (Ministère de l’Agriculture, France) guidelines.

### C9ORF72 knockdown validation

*C9orf72* mRNA was quantified by quantitative PCR (qPCR) in the cortex, spinal cord, and muscle. Primers and probe sequences are listed in Table 1.

**TABLE 1:** List of primers used for qPCR.

	Sense	Reverse
OraV1	TGGAGCAGGACATATTT GACGC	AGTGGGATCATCGTAA GGAAAGT
GAPDH	AGGTCGGTGTGAACGGATTTG	GGGGTCGTTGATGGCAACA
C9ORF72	CGCAGGACACCATCATCTAC	GGCTTCAAATGGAAGACCTG

### Behaviour tests

Prior to any behavior test, mice were allowed to acclimatize to the testing room for 20 min. Material was cleaned between each animal with Aniospray. Males were tested before females and the equipment was washed with water between testing groups. Standard procedures were used for each test. For each test, results from males and females were pooled after verifying that sex did not alter the results significantly. The sex ratio was balanced in each group: 50, 60, and 47% of males in the wild-type, miR-Scramble, and miR-C9orf72 groups, respectively.

### Histological analysis

Sections of paraffin-embedded brain and spinal cord were stained using standard protocols with antibodies against Iba1, GFAP, NeuN, TDP-43, p62, and SV2. Digital images were captured with an Axioscan slide scanner (Zeiss, Oberkochen, Germany) or with an Apotome (Zeiss, Oberkochen, Germany) at 1.5 and 2.0 mm from the interneural line. Whole mounts specimens of soleus, extensor digitorum longus (EDL) and diaphragm muscles were stained for AchR with TRITC-labeled α-bungarotoxin, for hNL168, SV2 and images were acquired with a confocal microscope (Olympus FV-1000, Tokyo, Japan). Neuronal loss quantification was done by counting the totality of neurons in frontal and motor cortices using the Stereo Investigator software (MBF Bioscience, Williston, ND, United States). Quantification of positive Iba1 and GFAP cells was done on images covering the totality of frontal and motor cortices. Non-punctate p62 staining larger than 2 μM was counted as p62 accumulation. Positive staining for cytoplasmic TDP-43 and accumulated p62 were counted in a total of 250 to 500 cells per structure and per animal; positive cells for cytoplasmic staining were normalized to the total number of TDP-43 positive cells. Lumbar motor neurons marked with Nissl staining were imaged using a Leica (Wetzlar, Germany) DM250 microscope (20x). Morphological endplate count was done on confocal images. All cells counts were done by an experimenter who was blind to the genotypes. Counts were reported to the surface area (mm^2^) using ImageJ software (National Institutes of Health, Bethesda, MD, USA). In each group, an equal number of males and females were analyzed.

### Statistical analysis

For behavioral analyses, intergroup differences were evaluated by non-parametric Kruskal-Wallis tests followed by post-hoc Dunn Tests. Quantification analyses were evaluated with parametric two-way ANOVAs followed by Tukey’s multiple comparison tests, after verifying normality with D’Agostino and Pearson omnibus normality tests. Electrophysiological analyses were performed with paired-*t*-tests. All tests were performed using Prism software (Graphpad Software Inc). Values of *p* < 0.05 were considered statistically significant. All data are presented as means ± SEM.

## Results

### C9ORF72 patients’ haploinsufficiency is mimicked by genetic knockdown of the mouse C9orf72 gene

**FIGURE 1:**
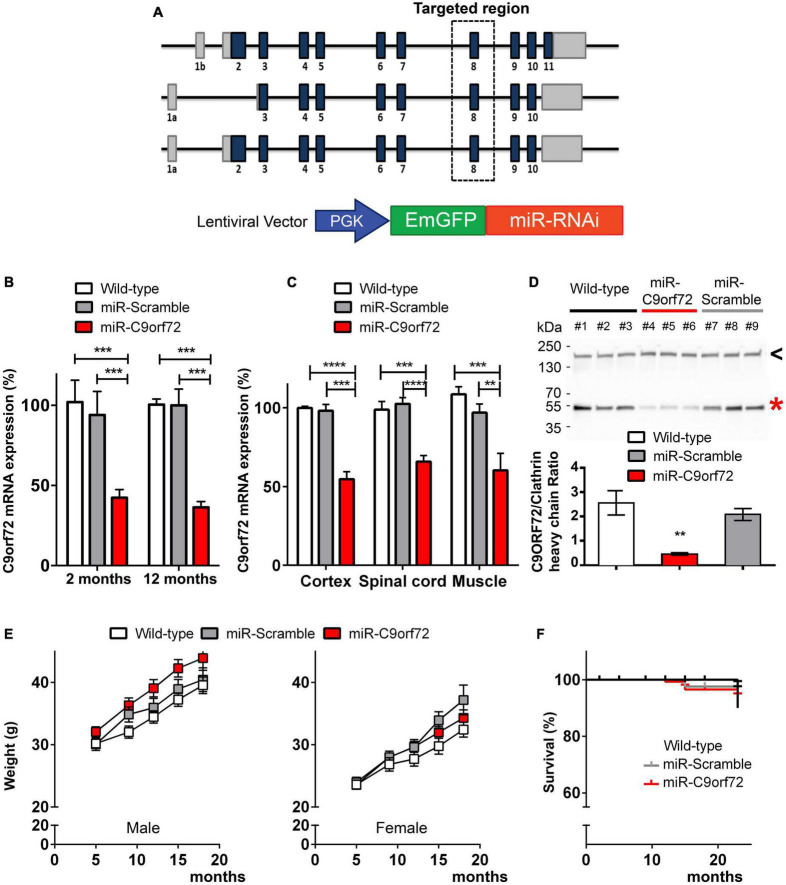
Generation of *C9ORF72* knockdown mice. **(A)** Schematic representation of C9orf72 transcripts in the mouse and the lentiviral vector used to perform transgenesis. MiR-C9orf72 targeted region is denoted by a square zone. **(B)** Relative expression of C9orf72 mRNA in transgenic mice cortex, at 2 and 12 months measured by qPCR. (wild-type *n* = 3; miR-Scramble *n* = 3, c9 *n* = 6). **(C)** Relative expression of C9orf72 mRNA in transgenic mice cortex, spinal cord, and muscle measured by qPCR at 23 months (wild-type *n* = 10; miR-Scramble *n* = 12, c9 *n* = 34). **(D)** Levels of mouse C9ORF72 protein orthoog in the cortex of miR-C9orf72, miR-Scramble, and wild-type mice. The expression of C9ORF72 (red asterisk) was quantified by densitometric analysis of western blots and normalized to clathrin heavy chain (black arrowhead). The positions of the molecular weight marker are indicated on the left in kDa. **(E)** Weight curve of males (left) and females (right) miR-C9orf72 mice compared to controls (wild-type *n* = 10; miR-Scramble *n* = 12, miR-C9orf72 n = 34). **(F)** Survival curve of C9ORF72 deficient and control animals up to 24 months (wild-type *n* = 10; miR-Scramble *n* = 12, miR-C9orf72 *n* = 34). Error bars represent SEM; *p < 0.05, **p < 0.01, ***p < 0.001, ****p < 0.0001.

We designed a miR-RNAi sequence targeting a region located in exon 8 of the *C9orf72* mouse ortholog which is common to all transcript variants, to knockdown the expression of C9ORF72 (Figure 1A), and thus mimic the downregulation effect seen with the human mutation. A lentiviral vector containing this miR-RNAi sequence and a GFP reporter gene under the control of a ubiquitous promoter (phosphoglycerate kinase, PGK, Figure 1A) was injected in mouse oocytes to generate lentitransgenic animals with a range of *C9orf72* knockdown (miR-*C9orf72*), as has been previously observed in patients (DeJesus-Hernandez et al., 2011; Ciura et al., 2013). Similarly, transgenic mice expressing a scramble miR-RNAi were generated as control animals (miR-Scramble). An initial verification was performed at E14.5 confirming lentiviral expression ubiquitously in transgenic mice (Supplementary Figure 1A). C9ORF72 deficient animals present a decrease of *C9orf72* transcripts of approximately 50% which is stable during the lifetime of the animal and observed in all regions of interest (Figures 1B, C). This RNA decrease translates at the protein level (Figure 1D) similarly to what we observed previously in frontal cortices of patients (Viodé et al., 2018). Thus, these results demonstrate an ubiquitous knockdown of C9ORF72 corresponding to the decreased expression seen in patients cortices (DeJesus-Hernandez et al., 2011; Belzil et al., 2013; Ciura et al., 2013; Waite et al., 2014; Frick et al., 2018; Viodé et al., 2018).

C9ORF72 deficient mice were comparable to controls in viability, appearance, fertility, and weight (Figure 1E). Finally, survival was not significantly affected in these animals, as they were still alive after 23 months of age (Figure 1F).

### C9ORF72 deficient animals present an accumulation of autophagy/lysosomal proteins, cytoplasmic TDP-43 and decreased density of synaptic vesicle protein 2 in the cortex

**FIGURE 2:**
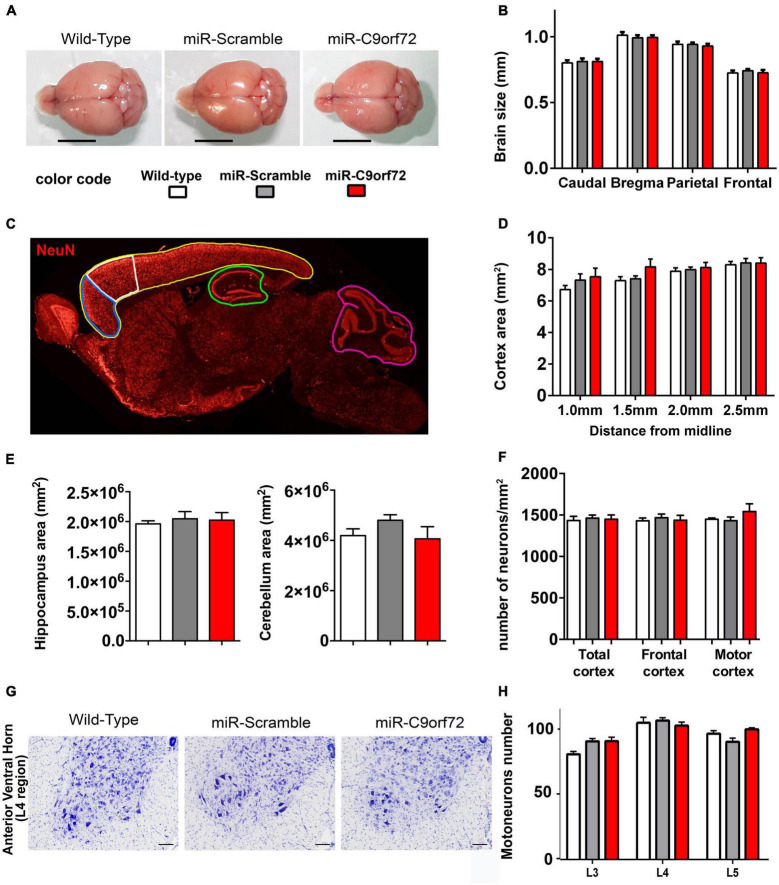
*C9ORF72* deficient mice do not present brain atrophy or neurodegeneration. **(A)** Representative examples of adult brains of miR-C9orf72, miR-Scramble and wild-type mice at 18 months. Scale bar = 5 *mm.***(B)** Macro-measurements of brain width at caudal, bregma, parietal and frontal positions (wild-type n = 4; miR-Scramble n = 4, miR-C9orf72 n = 6). **(C)** Areas of the brain analyzed: total cortex (yellow), frontal cortex (blue), motor cortex (white), hippocampus (green), and cerebellar (pink) (wild-type n = 4; miR-Scramble n = 4, miR-C9orf72 n = 6). **(D)** Quantification of the total cortical surface between 1 and 2.5 mm from interaural line. **(E)** Quantification of the total hippocampal (left) and cerebellar (right) surface at 2 mm from interaural line. **(F)** Quantification of NeuN-positive cells in the whole cortex area (total cortex) and specifically in the frontal cortex and motor cortex, 2 mm from interaural line. wild-type *n* = 4; miR-Scramble *n* = 4, miR-C9orf72 *n* = 6. **(G)** Anterior ventral horn sections of C9ORF72 deficient mice and controls at 22 months of age stained with Cresyl Violet (Nissl staining). Scale bar = 200 μ m. **(H)** Quantification of motor neurons in L3, L4, and L5 sections (wild-type *n* = 4; miR-Scramble *n* = 4, miR-C9orf72 *n* = 6). Error bars represent SEM.

We next evaluated whether decreased expression of C9ORF72 caused neuronal loss or degeneration resembling the neurodegenerative state of patients’ brains. At 23 months of age, miR-*C9orf72* mice did not present brain atrophy (Figures 2A, B). There was no marked reduction of the whole cortical, hippocampal or cerebellar areas (Figures 2C–E). The number of NeuN-positive neurons in total cortex, frontal cortex, and motor cortex was similar in C9ORF72 deficient mice when compared to controls (Figure 2F and Supplementary Figure 1B). Similarly, the spinal cord of miR-*C9orf72* mice appeared intact and no difference in motoneurons numbers or morphology was observed between groups at 23 months of age (Figures 2G, H).

**FIGURE 3:**
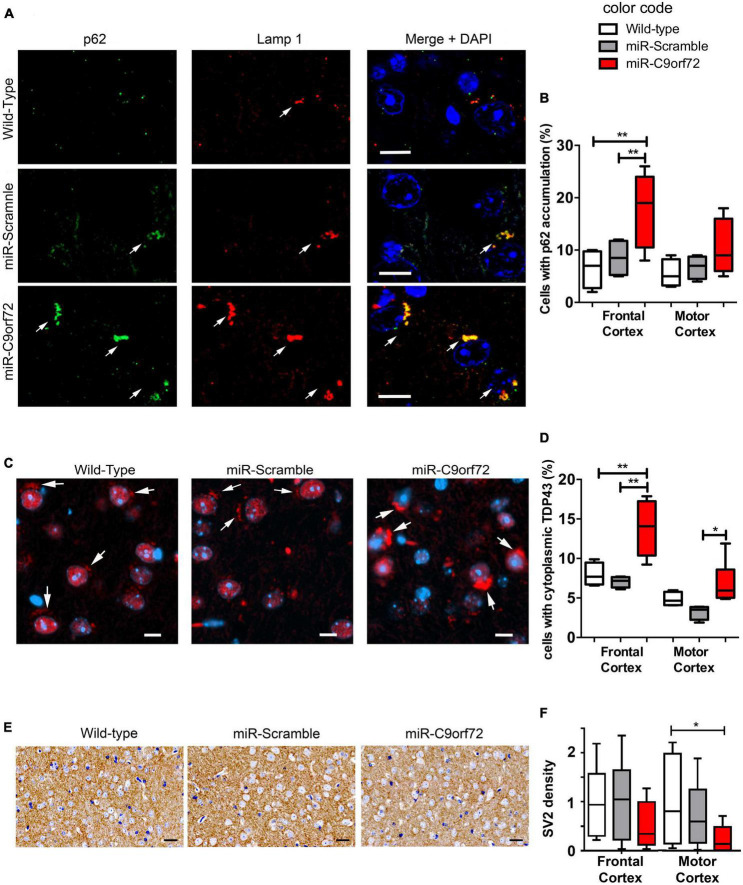
*C9ORF72 knockdown causes p62 and cytoplasmic TDP-43 accumulation in the cortex*. **(A)** Immunofluorescence co-staining of the frontal cortex from wild-type, miR-Scramble mice and miR-C9orf72 using anti-p62 (green) and anti-Lamp1 (red). Accumulation of p62 positive large structures that stain positive for Lamp1 is observed in C9ORF72 deficient mice (arrows). Scale bar: 10 μm. **(B)** Quantification of cells presenting p62 accumulation in the frontal and the motor cortex of controls and C9ORF72 deficient animals. **(C)** Immunofluorescence staining of TDP-43 (red) in sagittal sections of the cortex from wild-type, miR-Scramble mice and miR-C9orf72. Cytoplasmic structures that stain positive for TDP-43 are indicated by arrows. Scale bar: 10 μm. **(D)** Quantification of cells presenting cytoplasmic TDP-43 accumulation in the frontal and the motor cortex of controls and C9ORF72 deficient animals. **(E)** 3,3′-diaminobenzidine (DAB) staining of SV2 positive synapses in in sagittal sections of the cortex from wild-type, miR-Scramble mice, and miR-C9orf72. Scale bar: 20 μm. **(F)** Quantification of the normalized density of SV2 staining in the frontal and the motor cortex of controls and C9ORF72 deficient animals (the average density of SV2 staining in wild-type animals was used to nomalize all densities). For all experiments, wild-type and miR-Scramble *n* = *4; miR-C9orf72 n* = *6*. Error bars represent SEM; *p < 0. 05, **p < 0.01.

Absence of overt neurodegeneration is not uncommon in mouse models of FTD/ALS and was often observed in other C9ORF72 loss of function models as well as in gain of function models (Balendra and Isaacs, 2018; Braems et al., 2020). However, neuropathological signs of neuronal stress and dysfunction characteristic of C9-FTD/ALS are more easily modeled in mice and may thus be present. Astrocyte and microglia activation is implicated in the onset and progression of neurodegeneration in both ALS and FTD (Radford et al., 2015), but at the histological level C9ORF72 deficient animals showed no signs of increased glial activation at 23 months. The number or GFAP positive cells and Iba-1 positive cells were similar to controls (Supplementary Figures 1C–F) and there was no sign of peripheral inflammation like splenomegaly (Supplementary Figure 1G), previously observed in *C9orf72* knockout animals (Atanasio et al., 2016; Burberry et al., 2016; Jiang et al., 2016; O’Rourke et al., 2016). The C9ORF72 protein is involved at different levels of the endosomal, lysosomal and autophagy pathway (Levine et al., 2013; Amick et al., 2016; Blokhuis et al., 2016; Sellier et al., 2016; Sullivan et al., 2016; Webster et al., 2016; Xiao et al., 2016; Yang et al., 2016; Jung et al., 2017; Liang et al., 2019; Staats et al., 2019; Shao et al., 2020) which plays a key role in protein metabolism and recycling in neurons and is strongly involved in TDP-43 degradation (Filimonenko et al., 2007; Ju et al., 2009; Urushitani et al., 2010; Wang et al., 2012; Barmada et al., 2014; Scotter et al., 2014). In patients, cytoplasmic accumulation of p62 and TDP-43 are a major hallmark of C9-FTD/ALS pathology (Neumann et al., 2006; Mackenzie et al., 2014). Regarding p62, C9ORF72 deficient animals presented twice as many cells containing p62 accumulations in the frontal cortex when compared to controls at 23 months (Figures 3A, B). Furthermore, these p62 structures stained positive for lysosomal marker Lamp1 in C9ORF72 deficient animals (Figure 3A). Similarly, to assess TDP-43 pathology, we quantified cytoplasmic accumulation of TDP-43 in both frontal and motor cortices. As previously observed in aged animals (Thammisetty et al., 2018) cytoplasmic staining for TDP-43 was observed in some cells in the cortex of control mice, but TDP-43 positive structures appeared more compact and their number was largely increased in the frontal cortex of C9ORF72 deficient animals (Figures 3C, D and Supplementary Figure 2A). Interestingly, TDP-43 cytoplasmic accumulations were more prevalent in layers 5–6 of both cortices compared to layers 2–3 (Supplementary Figure 1H). 48% of these TDP-43 cytoplasmic accumulations were also positive for p62 (Supplementary Figure 2B). Synaptic impairment is also an important pathological mechanism is FTD/ALS and both gain and loss of function mechanisms of the C9ORF72 mutation can lead to synapse loss in a diversity of models (Choi et al., 2019; Lall et al., 2021; Nishimura and Arias, 2021; Huber et al., 2022a,b). We measured the density of the synaptic marker Synaptic Vesicle Protein 2 (SV2) in the frontal and motor cortices of the C9ORF72 deficient mice and observed that it was markedly decreased relatively to control animals (Figures 3E, F). Moreover, immunoblot analyses showed that SV2 was globally decreased in the cortex of C9ORF72 deficient mice (Supplementary Figures 2C, D). Taken together, these data show that C9ORF72 deficiency alone is not sufficient to trigger neurodegeneration or glial activation in the brain of mice, but causes cellular pathology characteristic of C9-FTD/ALS.

### C9ORF72 knockdown causes altered social interaction and depression-like behavior in mice

Frontotemporal dementia symptoms are very complex and can hardly be fully recapitulated in murine models. However, most cases of C9-FTD present the behavioural variant of the disease (bv-FTD), for which some characteristic behaviors can be evaluated in mice and some phenotypes may be considered FTD-like. MiR-*C9orf72* mice were subjected to a battery of behavioral tasks at 2, 5, 9, 12, 15, and 18 months of age in order to determine whether decreased expression of C9ORF72 caused such phenotypes, in particular anxiety-like or depression-like behaviors and alterations of social interaction, as was the case in other genetic FTD models (Yin et al., 2010; Roberson, 2012; Filiano et al., 2013).

**FIGURE 4:**
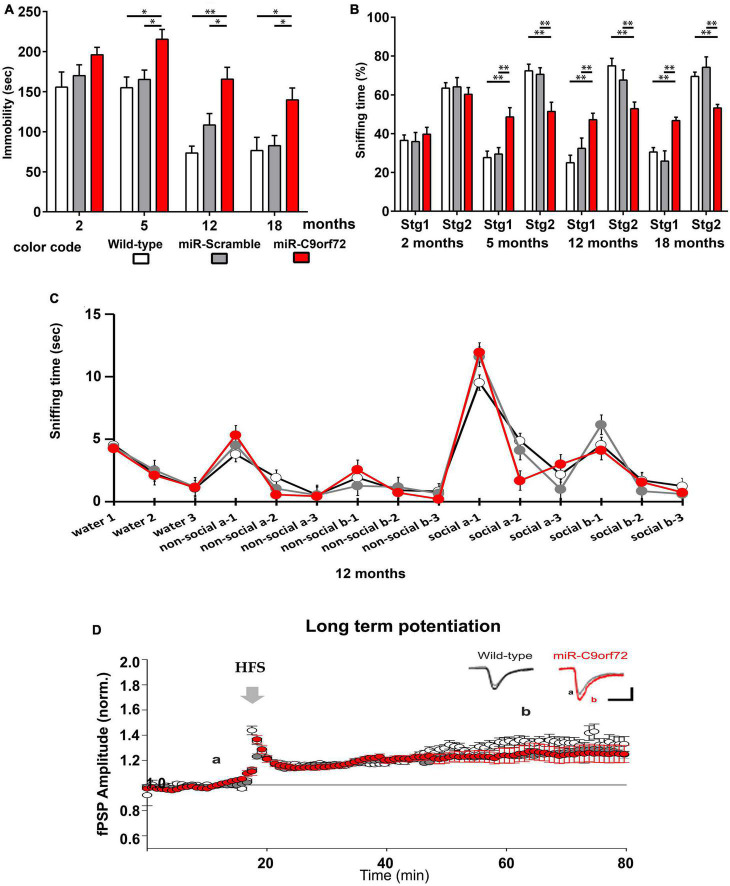
miR-C9orf72 mice develop FTD-like behaviors. **(A)** Forced swim assessment for depression-like behavior at 2, 5, 12, and 18 months (wild-type n = 10; miR-Scramble n = 12, miR-C9orf72 n = 34). Depression-like behavior is measured as the time of immobility in the water tank. **(B)** Three-chamber test for social interaction and social novelty at 2, 5, 12, and 18 months (wild-type n = 10; miR-Scramble n = *12, miR-C9orf72 n* = 34). Social exploration is quantified as time spent sniffing an already known mouse (stg1) or a novel mouse (stg2). **(C)** Olfactory habituation/dishabituation test at 12 months used to measure if animals can differentiate between same and different odors, social and non-social. For all mice, consecutive presentations of the same odor resulted in decreased investigation of the smell showing habituation. Sniffing time increased again each time the subject was introduced to a new smell and social cues elicited a higher response than non-social ones as expected demonstrating normal olfaction and memory (wild-type n = 6; miR-Scramble n = 6, miR-C9orf72 n = 7). **(D)** Study of long-term potentiation in the CA1 area. High frequency stimulation (HFS; 2 × 100 Hz, 1 s) of CA3 Schaffer collaterals induced a long-term potentiation (LTP) of the CA1 post-synaptic field potential (fPSP) in all animals tested. The LTP amplitude was similar between the miR-C9orf72 and control animals. At the top right, representative sample traces from an electrode before (gray) and after the LTP induction in the miR-C9orf72 (red) and the WILD-TYPE (black) (wild-type n = 6; miR-Scramble n = 6, miR-C9orf72 n = 7). Error bars represent SEM; *p < 0.05, **p < 0.01.

From early stages of bv-FTD pathology, patients can present apathy, a loss of initiative and motivation that is common to both FTD and depression. The forced swim test has been classically used to measure depression-like behavior in mice and can detect apathetic behavior (Porsolt, 2000). In this test, C9ORF72 deficient animals presented strikingly longer periods of immobility at 5, 9, 12, 15, and 18 months of age when compared to controls (Figure 4A). However, when accounting for exploration or anxiety-like behavior in a novel-environment with the open-field test and the dark and light chamber test, no difference was observed among groups (Supplementary Figures 3A, B).

Progressive deterioration in social function is another characteristic of bv-FTD (Burrell et al., 2016). Altered social interactions in mice represents a phenotype reminiscent of social deficits observed in FTD patients that can be investigated using the three-chamber test for sociability and social novelty preference (Yang et al., 2011). During the sociability session, C9ORF72 deficient mice all preferred the mouse rather than the object, similarly to controls (Supplementary Figure 3C). However, they proved unable to distinguish between the “novel mouse” and the “known mouse” in the social novelty preference session, from 5 months of age onward through all subsequently tested ages (Figure 4B), revealing a profound dysfunction of social behavior. To make sure that this result really reflected abnormal social functions we ran a number of complementary tests to exclude confounding effects due to olfactory defects or memory impairment. We verified olfactory abilities with the habituation/dishabituation test, which showed that miR-*C9orf72* mice were perfectly able to identify a new odor and to differentiate between a social odor and a non-social one (Figure 4C). Hippocampal memory also appeared unaffected as no phenotype was detected using the Morris-water maze test (Supplementary Figures 3D, E), and long-term potentiation (LTP) and synaptic transmission at the Schaffer collateral-CA1 synapse was fully functional and similar between C9ORF72 deficient mice and controls (Figure 4D and Supplementary Figures 3F–H).

These observations reveal for the first time that decreasing C9ORF72 *in vivo* in mice can lead to apathetic or depression-like behaviors, and very specifically alters social interaction at as early as 5 months of age.

### C9ORF72 deficient mice present mild strength loss and neuromuscular junction abnormalities but no motor neuron disease

**FIGURE 5:**
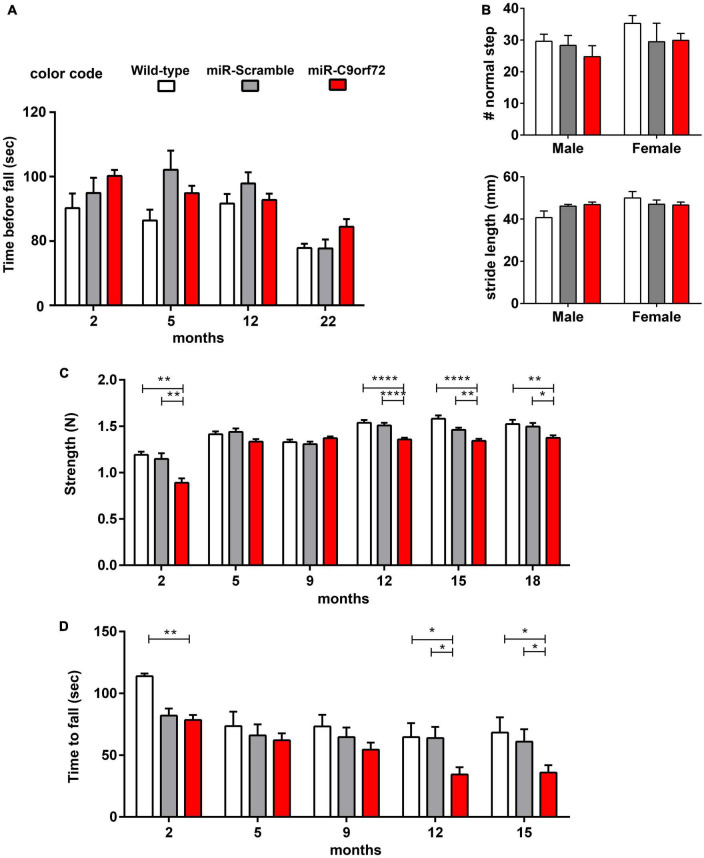
Age-dependent strength loss but absence of motor deficits in C9ORF72 deficient mice. **(A)** Rotarod testing of C9ORF72 deficient mice at 2, 5, 9, 12, and 22 months (wild-type n = 10; miR-Scramble n = 12, miR-C9orf72 n = 34). No differences of sensorimotor coordination, or motor learning were observed across trials in young or aged animals. **(B)** Quantification of normal step number and stride length by Treadmill. No alteration could be seen with C9ORF72 deficient animals (wild-type n = 10; miR-Scramble n = 12, miR-C9orf72 n = 34). **(C)** Hind limb grip strength measured at 2, 5, 9, 12, 15, and 22 months (wild-type n = 10; miR-Scramble n = 12, miR-C9orf72 n = 34). **(D)** Hanging wire test measuring time to fall at 2, 5, 9, 12, and 15 months (wild-type n = 10; miR-Scramble n = 12, miR-C9orf72 n = 34). Error bars represent SEM; *p < 0.05, **p < 0.01, ****p < 0.0001.

Patients bearing *C9ORF72* HRE can present FTD, ALS, or both. To test whether ALS-like anomalies arise when knocking down C9ORF72, despite the absence of motoneuron degeneration (Figures 2G, H) we assessed motor performance, strength, and neuromuscular transmission in our mouse model. Using the accelerating rotarod, C9ORF72 deficient mice demonstrated normal balance and coordination (Figure 5A). To further assess miR-*C9orf72* mouse locomotion, video footage of the three groups walking on a treadmill was used for precise gait analysis. Gait traces were produced from which step, stance and swing duration were analyzed. A regularity index was then computed. No major differences were observed in gait coordination and execution of step timing (Figure 5B). No differences in traveled distance or velocity were either observed in the Open-Field test (Supplementary Figure 4A). Next, mice were tested for muscle strength using the grip test, during which the peak of hindlimb maximal force is measured. Remarkably, miR-*C9orf72* mice presented a deficiency that appeared with aging starting at 12 months (Figure 5C). Similar results were observed with the hanging wire test, confirming the observed effect (Figure 5D). We also noted that C9ORF72 deficient mice appeared slower to reach their adult peak of force compared to controls in the grip test, but the hanging wire test showed that they were similar to miR-Scramble controls at that age (Figures 5C, D).

**FIGURE 6:**
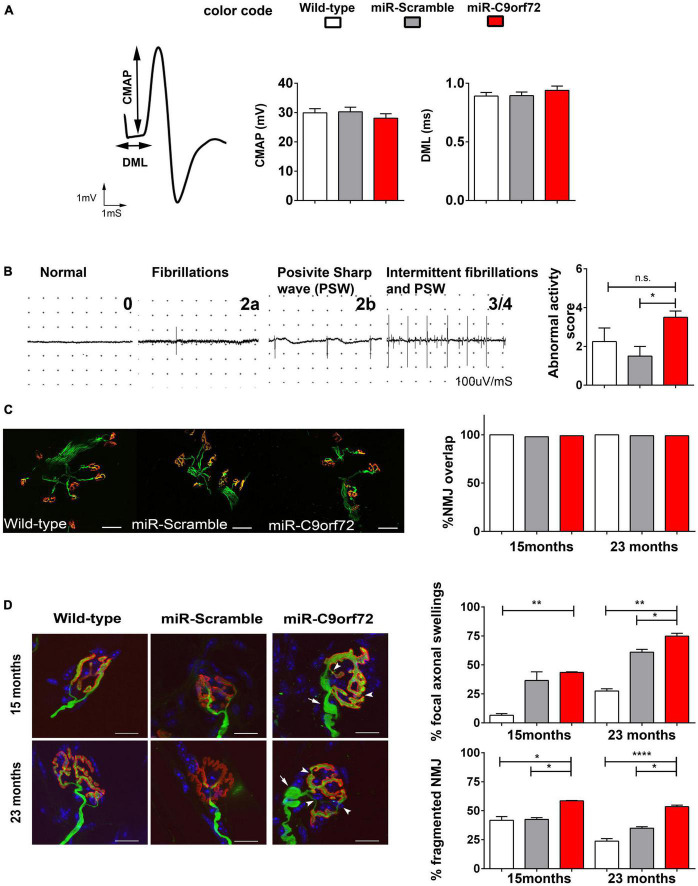
C9ORF72 deficient mice show evidence of mild neuromuscular junction deficits. **(A)** Representative tracings of evoked Compound Muscle Action Potentials (CMAPs) at 20 months in the gastrocnemius muscle after stimulation of the sciatic nerve. Distal Motor Latency (DML), expressed in ms, was determined by the time between the stimulus and the time to onset of a negative peak in the CMAP as shown by arrows. Both graphs present DML in ms as means ± SEM and CMAP in millivolts, as means ± SEM for wild-type, miR-Scramble and miR-C9orf72 groups (*n* = 8). **(B)** Representative tracings of abnormal activities used to score the EMG abnormal activities at 20 months: normal activity (0)/presence of abnormal activities (1) fibrillation (2a)/positive sharp wave (2b) and intermittent activities (3). The graph present EMG abnormality score rating 0 to 4 (wild-type *n* = 8; miR-Scramble *n* = 8, miR-C9orf72 *n* = 8). **(C)** Analysis of synaptic contact in diaphragm muscle of wild-type, miR-Scramble and miR-C9orf72 mice. Whole-mount preparations stained with α-bungarotoxin for acetylcholine receptors (nAChR) in red and with anti-neurofilament (NF) and anti-SV2 antibody for axons and synaptic vesicles in green. NMJ overlaps quantification shows no differences between synaptic contacts in miR-C9orf72 when compared to controls. Scale bar = 10 μm. **(D)** Morphological study of NMJ in Soleus muscle of wild-type, miR-Scramble and miR-C9orf72 mice. MiR-C9orf72 mice present abnormal focal axonal swellings (arrows). The histograms represent quantification of both abnormalities expressed as a percentage of the 224, 260, and 243 NMJs analyzed in miR-C9orf72, wild-type and miR-Scramble mice, respectively. Scale bar = 10 μm. Error bars represent SEM; n.s. non-significant; *p < 0.05, **p < 0.01, ****p < 0.0001.

We next used electromyogram (EMG) recordings to determine whether neurophysiological abnormalities occur in the peripheral nervous system. Compound muscle action potential (CMAP) amplitude as well as distal motor latency (DML) were normal, thus excluding major motor conduction changes in miR-*C9orf72* mice (Figure 6A). Similarly, muscle fibers did not present changes at the histological level (Supplementary Figure 4B). Reduced muscle strength may result from motoneuron loss, neuromuscular transmission failure, or muscle atrophy. As motoneuron loss, motor conduction changes or muscle atrophy were not observed, we looked into other possible mechanisms. As neuromuscular junction innervation can be strongly affected in ALS (Dupuis and Loeffler, 2009), we looked for signs of degeneration and/or muscle atrophy. Muscle fibrillations were more frequently observed in miR-*C9orf72* mice compared to miR-Scramble mice (Figure 6B), which suggested possible events of muscle denervation. We then studied individually the neuromuscular junction (NMJs) at the morphological level. Nerve terminals and motor plates were perfectly colocalized, excluding massive denervation events (Figure 6C). However, we observed a mild yet significant remodeling of pre- and post-synaptic compartments (Figure 6D). Unlike wild-type and miR-Scramble mice in which we observed typical fork-shaped nerve terminals innervating well-defined post-synaptic pretzel organization of the acetylcholine receptors (AChR), the miR-*C9orf72* mice presented pre- and post-synaptic defects in all tested muscles at 15 and 23 months of age (Figure 6D). Fragmentation of post-synaptic gutters and focal pre-terminal axonal swellings were most frequently seen in miR-*C9orf72* mice when compared to controls (Figure 6D).

Thus, in agreement with previous reports (Koppers et al., 2015; Atanasio et al., 2016; Burberry et al., 2016; Jiang et al., 2016; O’Rourke et al., 2016; Sudria-Lopez et al., 2016; Sullivan et al., 2016; Ugolino et al., 2016; Zhu et al., 2020) C9ORF72 decrease does not cause a motor phenotype in mice. However, we show that it can cause deficits in muscle strength with aging and light NMJ structural alterations, signaling late distal axonal suffering.

## Discussion

We have used a constitutive and ubiquitous knockdown to reproduce in mice the decrease in C9ORF72 RNA and protein expression observed in patients. Using these miR-*C9orf72* mice, we conducted an extensive behavioral, histological and neurophysiological characterization over 24 months, which allowed us to investigate in more detail alterations that can arise from reduced C9ORF72 in the brain, spinal cord and muscles over time. In agreement with previous work (Koppers et al., 2015; Atanasio et al., 2016; Burberry et al., 2016; Jiang et al., 2016; O’Rourke et al., 2016; Sudria-Lopez et al., 2016; Sullivan et al., 2016; Ugolino et al., 2016; Zhu et al., 2020), there was no overt neuronal loss in our mice, nor did they develop any locomotor dysfunction or motoneuron loss. However, subtle strength decrease, NMJ alterations, and distal axonal swellings developed in aged animals. They also exhibited abnormal accumulations of key proteins involved in the autophagy/lysosomal functions, which are commonly altered in *C9orf72* KO animals (Koppers et al., 2015; Atanasio et al., 2016; Burberry et al., 2016; Jiang et al., 2016; O’Rourke et al., 2016; Sudria-Lopez et al., 2016; Sullivan et al., 2016; Ugolino et al., 2016; Zhu et al., 2020). Recently, alterations of the endosomal-lysosomal pathway were also reported in haploinsufficient *C9orf72* mice supporting our findings (Staats et al., 2019). Interestingly, we found an increased number of neurons with cytoplasmic TDP-43 deposits, as had previously been seen in C9ORF72 loss of function cellular models (Sellier et al., 2016), and which might represent an early pathological event in the process leading to TDP-43 accumulation and aggregation. Previous studies on *C9orf72* knockout animals did not report a similar pathology, but TDP-43 mislocalization was either investigated in much younger animals or not quantified (Koppers et al., 2015; Burberry et al., 2016; O’Rourke et al., 2016). Since we observed it only in maximum 15–20% of the frontal cortex neurons, it may have been previously missed. In parallel, there was a decrease in the cortical density of the synaptic marker SV2, suggesting a decrease in the number of functional synapses in FTD/ALS related regions. Contrasting with the absence of motor neuron disease, we observed that our miR-*C9orf72* mice presented social interaction deficits and apathy or depression-like behavior as early as at 5 months of age. Therefore, we show that a C9ORF72 deficiency in mice reflecting the decrease of C9ORF72 expression observed in patients is enough to trigger an FTD-like behavioral phenotype and some pathological alterations characteristic of C9-FTD/ALS. While it does not trigger an ALS-like phenotype, the deficiency also leads to mild alterations of the motor system with aging.

Since the discovery of the C9ORF72 mutation in FTD/ALS, the understanding of mechanisms that drive neurodegeneration in C9-FTD/ALS has raised a number of questions, none of them more debated than the relative roles of gain and loss of function effects. Models aiming to reproduce only one or the other have shed light on affected cellular pathways but have generally failed to fully mimic the disease or identify a main triggering effect (Balendra and Isaacs, 2018; Braems et al., 2020). On the one hand, RNA foci and DPRs seem to cause neurodegeneration and often lead to motor neuron disease when overexpressed (Chew et al., 2015; Zhang et al., 2016; Herranz-Martin et al., 2017; Schludi et al., 2017; Choi et al., 2019; Hao et al., 2019; LaClair et al., 2020), whereas most BAC models presenting RNA foci and RAN proteins at physiological levels do not fully develop neurodegeneration or FTD/ALS behavioral phenotypes (O’Rourke et al., 2015; Peters et al., 2015; Jiang et al., 2016; Liu et al., 2016; Mordes et al., 2020; Nguyen et al., 2020). On the other hand, knockout mice have been produced unraveling some of the functions of the C9ORF72 protein without extensively investigating behavioral characteristics of these mice, particularly in relation to FTD (Atanasio et al., 2016; Burberry et al., 2016; O’Rourke et al., 2016; Sudria-Lopez et al., 2016). Most interestingly, a thorough characterization was done in one study (Jiang et al., 2016) which showed that complete ablation of C9ORF72 resulted in sociability defects and late motor deficits. Lately, spatial memory impairment was also identified in aged C9ORF72^–/–^ mice (Lall et al., 2021). Therefore, it gradually appeared that the disease must result from a combination of events involving both gain and loss of function mechanisms (Balendra and Isaacs, 2018). Multiple evidence now supports the hypothesis that C9ORF72 haploinsufficiency synergizes with gain of function mechanisms to produce the FTD/ALS phenotype (Shao et al., 2019; Staats et al., 2019; Dong et al., 2020a,b; Zhu et al., 2020). Yet, how a decrease of C9ORF72 is going to alter the functions of the brain and spinal cord is still largely unknown.

In the first published models of C9ORF72 loss of function in mice, animals developed neither locomotor defects or motor neuron loss, or showed subclinical signs of degeneration (Lagier-Tourenne et al., 2013; Koppers et al., 2015). C9ORF72 reduction was, however, either exclusively neuronal (Koppers et al., 2015), or was only transiently knocked-down (Lagier-Tourenne et al., 2013). Other full knockout mouse models revealed very mild motor deficits that were not observed in C9ORF72 ± mice (Atanasio et al., 2016; Jiang et al., 2016). These deficits, consisting of progressive hindlimbs weakness and reduced locomotor activity appeared between 10 and 15 months (Atanasio et al., 2016), as well as a slight decrease in the latency to fall on a rotarod after 12 months (Jiang et al., 2016), are globally in agreement with the observations in our knockdown mice. Indeed, the decreased strength in hindlimbs coupled to weakness when hanging to an inversed grid, and pre and post-synaptic alterations in the NMJ denotes subclinical suffering of the motor unit (Figures 5C, D, 6B–D). The observed focal axonal swellings in the miR-*C9orf72* mice might represent early signs of future and more pronounced axonal damages (Nikić et al., 2011; Duregotti et al., 2013). “Focal axonal degeneration” (FAD) is characterized by sequential stages, beginning with focal axonal swellings, progressing to axon fragmentation, and eventually leading to motor neuron death (Craner and Fugger, 2011; Nikić et al., 2011). Although our evidence and others demonstrate that C9ORF72 deficiency cannot by itself cause ALS, it was recently shown in mice and rats that decreasing C9ORF72 predisposes animals to the development of motor neuron disease (Shao et al., 2019; Dong et al., 2020a,b; Zhu et al., 2020). In human motoneurons derived from iPS cells from C9ALS patients, neurodegeneration was also dependent on haploinsufficiency (Shi et al., 2018). Therefore, despite the numerous evidences that C9ORF72 loss of function does not cause motor neuron disease without additional hits, targeting C9ORF72 haploinsufficiency early on in the disease course might be of strong therapeutic interest in C9ALS.

Regarding the role of C9ORF72 deficiency in the development of C9-FTD, there has unfortunately been too few investigations in animal models. Social interaction deficits, in particular, are a common phenotype in FTD mouse models (Yin et al., 2010; Roberson, 2012; Filiano et al., 2013) and were only investigated by Jiang et al. (2016) and in the present study. Nevertheless, social behavior incongruities are observed early on in both studies (5–6 months) suggesting that decreasing C9ORF72 in the mouse results consistently in social interaction deficits. Strikingly, in gain of function models, however, although also rarely explored, this behavior generally appears unaffected (Peters et al., 2015; Jiang et al., 2016; Balendra and Isaacs, 2018). One study (Chew et al., 2015) with strongly overexpressed transgenic HRE reported social behavior anomalies, regarding, however, the ability to differentiate a social stimulus from a non-social object. This is quite different from a blunting of social recognition, which affects the ability of mice to identify and remember conspecifics, a most interesting phenotype in relation to FTD. In our study, miR-*C9orf72* mice were perfectly able to distinguish between social and non-social stimuli in two different tests (Figure 4C and Supplementary Figure 2C) whereas social recognition was impaired both in our model (Figure 4B) and in the knockout mice analyzed by Jiang et al. (2016).

Several groups showed that complete ablation of *C9ORF72* causes mice to develop autoimmunity (Atanasio et al., 2016; Burberry et al., 2016; Jiang et al., 2016; O’Rourke et al., 2016). Heterozygous mice do not seem to suffer from this abnormal immune activation (Atanasio et al., 2016; Burberry et al., 2016; Jiang et al., 2016; O’Rourke et al., 2016; Zhu et al., 2020) and we did not detect glial activation either in our miR-*C9orf72* mice (Supplementary Figures 1C–F) nor splenomegaly (Supplementary Figure 1G). This type of effect has been observed with the FTD-causing gene progranulin (*GRN*). *GRN* knockout mice often present changes in behavior relevant to FTD with increased inflammatory and phagocytic responses (Yin et al., 2010; Lui et al., 2016) while heterozygous mice can have FTD-related behavioral deficits without major signs of inflammatory alterations (Filiano et al., 2013). In GRN-FTD as well as in C9-FTD/ALS there is a strong possibility that more subtle alterations of the immune response can generate a critical frailty and contribute significantly to the disease. The fact that we observe signs of synaptic impairments may be in line with this hypothesis since it was recently observed as a consequence of microglial dysfunction in *C9ORF72* knockout mice (Lall et al., 2021). It is intriguing to note that both C9ORF72 and PGRN depletion are capable of causing systemic immune dysfunction in mouse models and FTD-related behaviors without motor neuron disease.

Interestingly, C9ORF72 has been shown to form a complex with SMCR8 to regulate the autophagy/lysosomal pathway (Levine et al., 2013; Farg et al., 2014; Almeida and Gao, 2016; Amick et al., 2016; Blokhuis et al., 2016; Sellier et al., 2016; Sullivan et al., 2016; Webster et al., 2016; Xiao et al., 2016; Yang et al., 2016; Jung et al., 2017; Liang et al., 2019; Shao et al., 2020). The autophagy receptor p62, which aggregates in the brain of C9ORF72 expansion carriers, accumulated in the cortices of our mice and colocalized with lysosomes. This result shows that decreased expression of C9ORF72 is enough to dysregulate the autophagy/lysosomal pathway, as was also observed in the hippocampus of *C9orf72*^±^ mice by Staats et al. (2019). Most importantly, the study of SMCR8 knockout mice has revealed that they develop autoimmunity similarly to C9ORF72 deficient animals (Zhang et al., 2018; Liang et al., 2019), but they also display mild motor phenotypes (Zhang et al., 2018) resembling those observed in C9ORF72 knockout mice (Atanasio et al., 2016; Jiang et al., 2016) and in our model. In SMCR8 and in C9ORF72 deficient mice, promoting autophagy *via* MTOR inhibition could rescue the autoimmunity phenotype, definitely establishing a causative link between the cellular function of C9ORF72 and immune dysfunction (Shao et al., 2020). It is however, unknown if SMCR8 knockout animals present disturbances of social interaction and if this phenotype as well as the mild motor deficits could be rescued in C9ORF72 and SMCR8 deficient animals by injecting MTOR inhibitors. The PIKfyve inhibitor apilimod was shown to rescue the endo-lysosomal impairment and increased glutamate receptor levels caused by C9ORF72 deficiency both *in vitro* and *in vivo* (Shi et al., 2018; Staats et al., 2019). Considering the hypothesis regarding the causes of the immune dysfunction in C9ORF72 knockout mice (Shao et al., 2020), it seems likely that apilimod or other PIKfyve inhibitors could rescue this phenotype as well. There is hope then that such molecules could rescue the behavioral phenotypes presented by C9ORF72 deficient mice, including our model, or even the SMCR8 knockout mice, offering a therapeutic strategy addressing the effects of C9ORF72 loss of function.

In conclusion, our study supports the hypothesis that decreased expression of C9ORF72 plays an important part in damaging target neuronal or glial cells involved in C9-FTD/ALS. Considering our results and those of others (Peters et al., 2015; Jiang et al., 2016; Balendra and Isaacs, 2018; Lall et al., 2021), we believe that C9ORF72 haploinsufficiency might be the main factor triggering the initiation of the FTD symptoms. However, most evidence suggests that additional stimuli or stresses such as those caused by RNA foci or DPRs are necessary to trigger the full neurodegenerative C9-FTD/ALS. In any case, in the light of recent data including the present work concerning the functions of C9ORF72 and the genetic evidence linking the autophagy/lysosomal pathway to FTD/ALS (Deng et al., 2017; Stamatakou et al., 2020), future therapies must be considered carefully. Strategies targeting the gain of function effects of the HRE should be designed to maintain or even increase in parallel the expression of the wild-type copy of *C9ORF72*.

## Data availability statement

The original contributions presented in this study are included in this article/Supplementary material, further inquiries can be directed to the corresponding author.

## Ethics statement

All animal experiments were approved by the institutional animal care and use committee CEEA—005 and in agreement with the European legislation N°2010/63 UE and national authority (Ministére de l’Agriculture, France) guidelines.

## Author contributions

M-BL-H, CL, SN, and ML designed the experiments and analyzed the data. SBa, BW, DR, JB, PF, PG, CD, and GD performed the experiments and analyzed the data. MF, DB, SD, and GD performed the experiments. MD, MN, GB, PR, MB, AC, and DS contributed to the methodology. IL and AB helped the design and coordinate the project. ML planned, designed, and coordinated the project. M-BL-H and ML wrote the manuscript. All authors contributed to the article and approved the submitted version.
