# Supplementary material for: C9ORF72 knockdown triggers FTD-like symptoms and cell pathology in mice
Source: Front Cell Neurosci. 2023 Apr 17;17:1155929. doi: 10.3389/fncel.2023.1155929 (PMC10149765; doi:10.3389/fncel.2023.1155929)
Supplement: Supplementary file 1 [file Data_Sheet_1.docx]

### SUPPLEMENTARY INFORMATION FOR

**C9ORF72 knockdown triggers FTD-like symptoms and cell pathology in mice.**

Maria-Belen Lopez-Herdoiza, Stephanie Bauché, Baptiste Wilmet, Caroline Le Duigou, Delphine Roussel, Magali Frah, Jonas Béal, Gabin Devely, Susana Boluda, Petra Frick, Delphine Bouteiller, Sébastien Dussaud, Pierre Guillabert, Carine Dalle, Magali Dumont, Agnes camuzat, Dario Saracino, Mathieu Barbier, Gaelle Bruneteau, Phillippe Ravassard, Manuela Neumann^,^, Sophie Nicole, Alexis Brice, Isabelle Le Ber, Morwena Latouche

#### Supplementary Experimental Procedures

##### Generation of C9ORF72 deficient mice and breeding

Following lentitransgenesis, GFP-positive male mice were backcrossed with females to obtain the tested F1 generation. Mice were kept on a 12h light/dark cycle with food and water available *ad libitum*. Transgene insertion was verified by PCR genomic DNA prepared from tail (Forward primer 5’-GACCACATGAAGCAGCACGACTTCT-3’ and Reverse primer 5’-TTCTGCTGGTAGTGGTCGGCGAGCT-3’).

##### C9ORF72 knockdown validation

*C9orf72* mRNA decrease was quantified by qPCR in cortex, spinal cord and muscle in animals using primers described in Table S1. Two controls genes were used to verify *C9orf72* mRNA reduction, GAPDH and OraV1. RNA was extracted using RNeasy Lipid-tissue Mini kit (Qiagen) from fresh frozen tissues and quality was verified with Bioanalyzer 2100 (Agilent).

Western Blot

C9ORF72 expression was analyzed by immunoblot of whole cortex RIPA lysates probed with GeneTex GTX634482 anti-C9ORF72 (1:500) and Abcam anti-Clathrin heavy chain (1:2000, ab21679) antibodies. Quantification of C9ORF72 signal intensities normalized to Clathrin heavy chain was done using the analysis software Image lab (Biorad). SV2 expression was analyzed by immunoblot of whole cortex RIPA lysates probed with an anti-SV2 antibody (1:1000, Hybridoma Bank, IA) and anti-β-Tubulin antibody (SV21:500, ab6046, Abcam)

###### Forced Swim test

To look for a depression-like behaviour, we tested the mice with the forced swim test. Mice were placed in a glass cylinder (height 25cm, diameter 14cm) filled with water (22ºC). Immobility was recorded in a single 7 min trial period. Immobility being the time the animal stayed without any active movement.

###### Social interaction test

Social interaction was tested with the classical three-chamber test. Mice were placed in a three-chamber rectangular apparatus (10cmx6cmx15cm per chamber) for a 3-stage testing protocol: Firstly, the animal freely explores the 3 chambers during a 10 min period. Then, a stranger mouse is placed in one of the lateral chambers. The time the tested mouse spends sniffing the unknown animal (stranger 1) is recorded during a 10-min period. Finally, a new mouse is placed in the opposite lateral chamber. The time the tested mouse spends sniffing this new animal (stranger 2) and the previous animal (stranger 1) is recorded for a 10-min period.

###### Habituation-Dishabituation odour assessment

To assess whether miR-*C9orf72* mice can smell and distinguish same and different odours, mice were examined with the habituation/dishabituation paradigm as previously described (Yang and Crawley, 2009). Habituation is defined by a progressive decrease in olfactory investigation (sniffing) towards a repeated presentation of the same odour stimulus. Dishabituation is defined by a reinstatement of sniffing when a novel odour is presented. Briefly, mice were confronted to two separate non-social odours and two separate social odours for a duration of 2 minutes. Time spent sniffing each odour was manually recorded. A pre-test acclimation phase presenting a neutral odour (water) was used as baseline. Each inter-trial had a 1 min duration.

###### Accelerated rotarod test

Motor coordination was evaluated on the accelerated rotarod (Columbus Instruments, OH, USA). Mice were placed on a revolving beam (diameter 4cm, width 8cm, height 38cm) for 4 successive trials for 2 days. The rod was accelerated gradually from 4 rpm to 28 rpm over 2 min. Latencies before falling were recorded.

###### Treadmill

Quantification of stride number, frequency, foot pressure and gait angle information was performed with the GaitScan software (CleversysInc©, VA). Mice were forced to pace at a 10cm/sec on a treadmill. A regularity index was computed accounting for motion variations.

###### Grip strength test

The pick of muscular force is measured with Columbus apparatus. Mice are placed in apparatus and rear limbs force is recorded in 4 separate trials. Resting time between trials was of 20 minutes.

###### Hanging wire

Mice are placed on a hanging grid (30cm height) suspended with all four limbs. Time before fall was recorded with a 2-min cutoff period in 3 separate trials. Resting time between trials was of 20 minutes.

###### Open Field test

To measure locomotion and anxiety-like behaviour we used the open field test. The open field apparatus (50x50 cm, wall height 50cm) was placed under the Topscan (CleversysInc©, VA) videotracking system. Mice were placed in one corner and the total distance moved was recorded in a 5-min session. The footage is then analyzed by an automated tracking system for the following parameters: total distance moved, velocity, and distance moved in a predefined zone at the center of the field. The “center zone” being more exposed, anxiety decreases the distance moved in the center.

###### Dark and Light Chamber

To measure social anxiety, mice were placed in a two-compartment (a dark one and a light one) chamber for duration of 5 minutes. Number of times and total time spent in the light chamber was recorded with Topscan (CleversysInc©, VA). Anxiety causes a decrease in number and duration of incursions in the light chamber.

###### Morris Water Maze test

To assess conserved spatial memory, we used the Morris water maze test. A 150cm diameter pool was filled with water (22ºC) made opaque by the addition of a non-toxic, odour-free paint. The escape platform was transparent plastic and was hidden 1cm under water surface in the target quadrant. The pool was placed in a room with several visual cues. Mice were placed next to and facing the pool wall successively in north, east, south and west positions. Latencies before reaching the platform were recorded in 4-trial sessions every day for 7 days using Anymaze tracking system (Stoeling ©). Whenever the mice failed to reach the escape platform within the 1-min cutoff period, they were placed on it for 8 sec. The probe trial was conducted on the 8^th^ day by removing the platform from the pool, placing the mouse next to and facing the north side. Time spent in the target quadrant was recorded in a 1-min trial.A visible platform control was performed afterwards, displacing it in all for quadrants and measuring the time the animal took to reach it.

##### Brain immunohistochemistry

Immunochemistry was performed on 5µm paraffin-embedded brain sections from 4 wild-type (2 males and 2 females), 4 miR-Scramble (2 males and 2 females) and 6 miR-*C9orf72* mice (3 males and 3 females). Briefly, hemi brains of wild type, miR-Scramble and miR-*C9orf72* mice (23 months) were post fixed in 4% PFA for 48h. Fixed hemi brains were then dehydrated in raising concentrations of ethanol. Dehydrated hemi brains were impregnated in paraffin and stored at room temperature until slicing. Leica apparatus was used to slice (5µm) brains in a sagittal plane. Slices were deparaffinated in decreasing ethanol baths. Epitope retrieval was performed in a citric acid bath warmed for10min at 600 watts in a microwave. Sections were blocked in 5% normal goat serum (NGS), 4 % BSA, PBS-Triton 0,2% for 1h at room temperature. Sections were incubated for 48h at 4°C with following primary antibodies in PBS containing NGS/4%BSA/0.2%Triton: rabbit polyclonal anti-Iba1 (1:500, Wako Chemicals, USA), mouse monoclonal anti GFAP (1:1000, Dako Agilent, USA), mouse monoclonal anti-NeuN (1:100, Millipore, USA), rabbit polyclonal anti-TDP 43 (1:200, Proteintech, USA), mouse monoclonal anti-p62 (1:100, Abcam, UK), SV2 antibody (1:500, Hybridoma Bank, IA). Secondary antibodies coupled to a fluorochrome were used to visualize immunostaining (goat monoclonal anti-mouse Alexa 555 1:1000, and goat monoclonal anti-rabbit Alexa 488 1:1000; Thermofisher Scientific, USA). Sections were incubated with secondary antibodies for 2 hours at room temperature and Dapi was used to stain nuclei. Sections were mounted using Aquapolymount (PolyScience, USA). Digital images were captured with slide scanner Axioscan (Zeiss, Germany) or with Apotome (Zeiss, Germany).

###### Neuronal loss quantification

Neuronal loss was quantified by counting the total number of NeuN positive neurons in frontal and motor cortex at 2.0mm from interaural line (from Paxinos Atlas) using Stereo Investigator software (MBF Bioscience, USA). A threshold was previously defined and all images were analyzed using the same parameters.

###### Quantification of microglia activation and GFAP-reactive cells

Cell count based on Iba-1 staining of microglial cells and GFAP+ astrocytes were performed on images encompassing the frontal and motor cortex. An experimenter who was blind to the genotype counted each positive cell. Number of cells counted was then reported to the surface area (mm^2^) measured using ImageJ software (National Institutes of Health, Bethesda, MD, USA).

###### Quantification TDP-43 and p62

Cells displaying cytoplasmic TDP 43 or p62 accumulation were quantified in five different images per animal at 20X by an experimenter who was blind to the genotype. The number of cells with cytoplasmic TDP 43 was then reported to the number of positive TDP-43 cells. The number of cells with cytoplasmic TDP 43 that was also positive for p62 was quantified. The number of cells with accumulated p62 was reported to the number of cells in the field (DAPI staining).

Quantification of SV2 density in the cortex

All SV2 stained slides were digitized at 400x magnification using ZEISS Axio Scan Z1 slides scanner. The highest focus homogeneity was achieved by using a stack of five optical sections and a subsequent application of extended depth of focus using the maximum intensity projection. Obtained whole slide images (WSI) were submitted to Visiopharm™ 2022.9 (Hoersholm, Denmark) for cell segmentation and signal intensity measurements. All WSI were analyzed using similar segmentation workflows. An integrated Bayesian linear classifier was trained to differentiate between the synaptic stains (SV2) and the non-stained tissue using normalized monochromatic brown (DAB) and blue (Hematoxylin) layers after a color deconvolution step. Obtained global stained area was subdivided in three categories (i.e., low, moderate and high) based on the intensity of the normalized DAB signal. For each animal, we calculated the ratio of the area of high positive SV2 staining and the total area in the frontal and the motor cortex. This SV2 staining density was then normalized to the mean density quantified in the wild-type animals.

##### Acute hippocampal slice preparations

Hippocampal slices were obtained from 18 months old wild-type (n=6), C9ORF72 deficient mice (n=7) and miR-Scramble (n=6) mice. Mice were anesthetized with pentobarbital (140mg/kg) and then perfused through the heart with an ice-cold “cutting” artificial cerebrospinal fluid (ACSF) solution containing (in mM) 75 Sucrose, 87 NaCl, 2,5 KCl, 7 MgCl_2_, 1,25 NaH_2_PO_4_, 26 NaHCO_3_, 0,5 CaCl_2_ and 25 glucose, saturated with 95% O_2_/5% CO_2_ (pH 7.2–7.3 and 315 mOsm/kg). Transversal slices (350 μm) were cut with a Leica VT1200S microtome (Leica Biosystems, Germany) at 0-2°C and recovered for 10 minutes in the same solution at 32°C and then for 1 hour at room temperature in the “recording” ACSF solution containing (in mM) 119 NaCl, 5 KCl, 2.5 CaCl_2_, 1.3 MgCl_2_, 9.3 KH_2_PO_4_, 25 NaHCO_3_, and 5 glucose, saturated with 95% O_2_/5% CO_2_ (pH 7.2–7.3 and 300 mOsm/kg). For MEA recordings, hippocampal slices were transferred to an MED64 probe (Alpha MED Scientific Inc., Japan) with an inter-electrode distance of 150 µm and continuously perfused (3 mL/min) with ACSF solution saturated with 95% O_2_/5% CO_2_ (pH 7.2–7.3 and 300 mOsm/kg) at 32 °C.

##### MEA recordings

Field post-synaptic potentials (fPSPs) were recorded in the stratum pyramidale and stratum radiatum layers of the CA1 hippocampal region using a multi-electrode array (MEA) system (MED64, Alpha MED Sciences). Precisely, fPSPs were evoked by stimulation of the Schaffer collaterals/commissural pathways with biphasic current pulses (200 µs). Input-output (I/O) curve were performed using a sequence of increasing stimulus amplitude from 10 to 100 μA. fPSPs were then evoked using a stimulation intensity which elicited 40-50% of the maximum response. Paired pulse facilitation (PPF) was measured using two stimulations with an inter-pulse interval varying from 20 to 200 ms. Percentage of facilitation was calculated by dividing fPSP slope elicited by the second pulse by the fPSP slope elicited by the first pulse. For LTP protocol, two stimulations with 50 ms inter-stimulus interval were applied at a frequency of 0.1Hz. LTP was induced by a high frequency stimulation (HFS) consisting of 2 trains at 100 Hz for 1 s delivered 5 s apart at 70–80% of the intensity that evoked the maximum fPSPs. Before HFS application, a stable baseline was first established for at least 15 min. To ensure input specificity and viability of slices, a second electrode was selected in the CA1-CA2 subicular region to stimulate a second input independent from the tetanized input. The magnitude of LTP was quantified as the percentage change in the fPSP initial slope (10–40%) and the peak-to-peak amplitude taken during the 45-60 min interval after HFS. Data were filtered at 1 kHz and digitized at 20 kHz and analyzed using Mobius software (Alpha Med Scientific). Additional data analyses were performed using Microsoft Excel and SigmaPlot. The experimenter was blind to the animal’s genotype throughout the experiments for both acquisition and analysis.

##### Spinal cord analysis

Spinal cord analysis was performed on 30µm paraffin-embedded sections from 4 wild-type, 4 miR-Scramble and 6 miR-*C9orf72* mice. Embedding protocol is identical to previously described protocol for brain sections. Spinal cord slices were deparaffinated in decreasing ethanol baths and lumbar motor neurons were marked with Nissl staining (Cresyl Violet). Motor neurons were quantified by an experimenter blind to genotype in Leica DM250 microscope (20X). Digital images were obtained with slide scanner Axioscan (Zeiss, Germany).

##### *In vivo* neurophysiological investigations

Neurophysiological investigations were performed on 20-month-old mice. Four male and four female mice were used. Mice were kept sedated with 2-3% isoflurane delivered *via* a mask during the full procedure. The body temperature was monitored with a rectal probe and maintained at 35-37°C by placing the animals on a warming pad. Bilateral hind limbs of all animals were examined using the Neuro-Mep-Micro electromyogram (EMG) apparatus (Neurosoft, Russia). A monopolar needle (Spesmedica, MN3512P150) was inserted subcutaneously at the base of the tail to ground the system. Spontaneous activities were searched for with a concentric needle electrode inserted in the *Gastrocnemius* (Technomed, TE/B50600-001). The signal was bandpass filtered 20 Hz to 10 KHz. We used an EMG abnormality score scale ranging from 0 (normal) to 4 (most severe) to estimate the extent of spontaneous activities (Evans et al., 2014). Briefly, our score takes into consideration the presence of spontaneous activities, fibrillations or positive sharp waves (PSWs), intermittent multiple fibrillations and PSWs, and their bilateral localization. To investigate the motor nerve conduction, the sciatic nerve was stimulated proximally at the sciatic notch and distally at the ankle with supramaximal stimuli of 0.2 ms through a needle electrode (Spesmedica, MN3512P150). Compound Muscle Action Potential (CMAP) was recorded by an active needle electrode inserted distally to the knee joint over the proximal portion of *Gastrocnemius* and a reference needle electrode inserted over the Achilles tendon. Distal Motor Latency (DML) was calculated from the latency of the stimulus to the onset of the negative peak of the CMAP. Baseline-to-peak amplitude measurements were used to compare wild-type, miR-Scramble and miR-*C9orf72* mice. The results from the left ant the right muscle evoked responses were measured and averaged. To assess for failure of neuromuscular transmission at the NMJ, the Repetitive Nerve Stimulation test (RNS) was performed as described in Echaniz-Laguna. A significant decrement was defined by a decrease of 10% between the ﬁrst and tenth CMAP negative peak amplitude response.

##### Morphological study of NMJ and muscle in mice

Soleus muscle, extensor digitorum longus (EDL) muscle and diaphragm were taken from mice at 5, 15 and 23 months of age after asphyxia in CO_2_. Whole mounts of leg specimens were fixed with 4% paraformaldehyde and diaphragms with 1% paraformaldehyde. All specimens were stained for acetylcholine Receptor (AChR) with TRITC-labeled α-bungarotoxin (1:1000, Molecular Probes, Leiden, The Netherlands), for neurofilaments with a 168 kDa neurofilament antibody (1:200, 2H3, Hybridoma Bank, IA) and for synaptic vesicles with a SV2 antibody (1:200, Hybridoma Bank, IA). Morphological analysis of the endplate was performed by confocal microscopy (Olympus FV-1000, Germany) and analyzed by ImageJ software (National Institutes of Health, Bethesda, MD, USA). For muscle structural analysis, fresh muscle specimens were snap-frozen in liquid nitrogen cooled isopentane for cryosection or fixed in 4% paraformaldehyde (PFA) diluted in PBS for whole-mount preparations. Transversal cryosections were stained with hematoxylin-eosin (H&E) and modified Gomori trichrome stains.

#### *
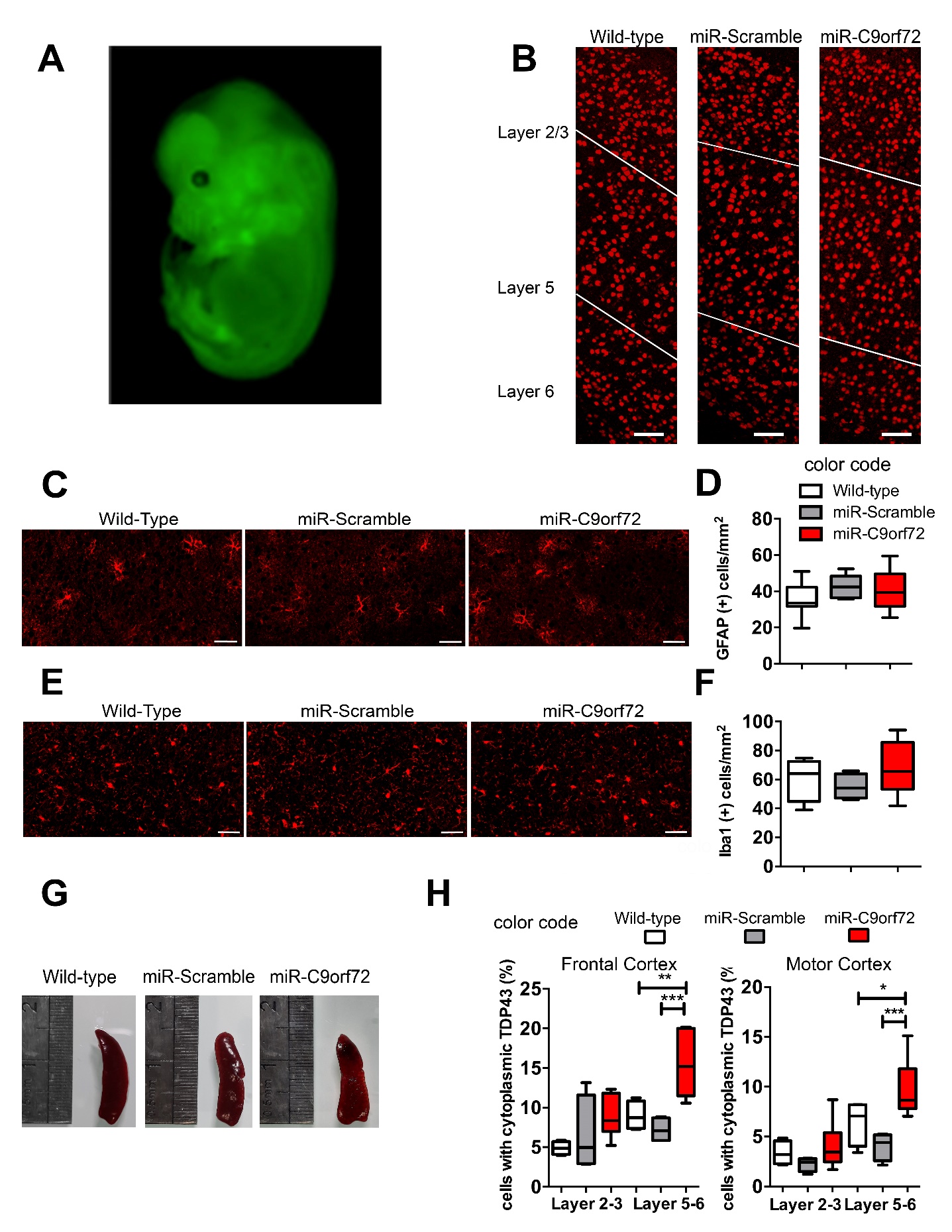
*

#### Supplementary Figure 1:

1. Ubiquitous transgene expression (visualized by GFP fluorescence) shown in mouse embryo.
2. Immunofluorescence staining of NeuN-positive cells in the frontal cortex 2 mm from interaural line. Scale bar: 100µm
3. Immunofluorescence staining of astrocytes in sagittal sections of the cortex from wild-type, miR-Scramble mice and miR-*C9orf72* using anti-GFAP (red). Scale bar: 50µm.
4. Quantification of GFAP positive cells in controls and C9ORF72 deficient animals.
5. Immunofluorescence staining of microglia in sagittal sections of the cortex from wild-type, miR-Scramble mice and miR-*C9orf72* using anti-Iba1 (red). Scale bar: 50µm.
6. Quantification Iba1 positive cells in the cortex of controls and C9ORF72 deficient animals.
7. Representative images of spleens from miR-*C9orf72*, miR-Scramble and wild-type mice.
8. Quantification of cells presenting cytoplasmic TDP-43 accumulation in Layers 2-3 and Layers 5-6 of the frontal and the motor cortex of controls and C9ORF72 deficient animals.

Error bars represent SEM; *p<0.05, **p<0.01, ***p<0,001


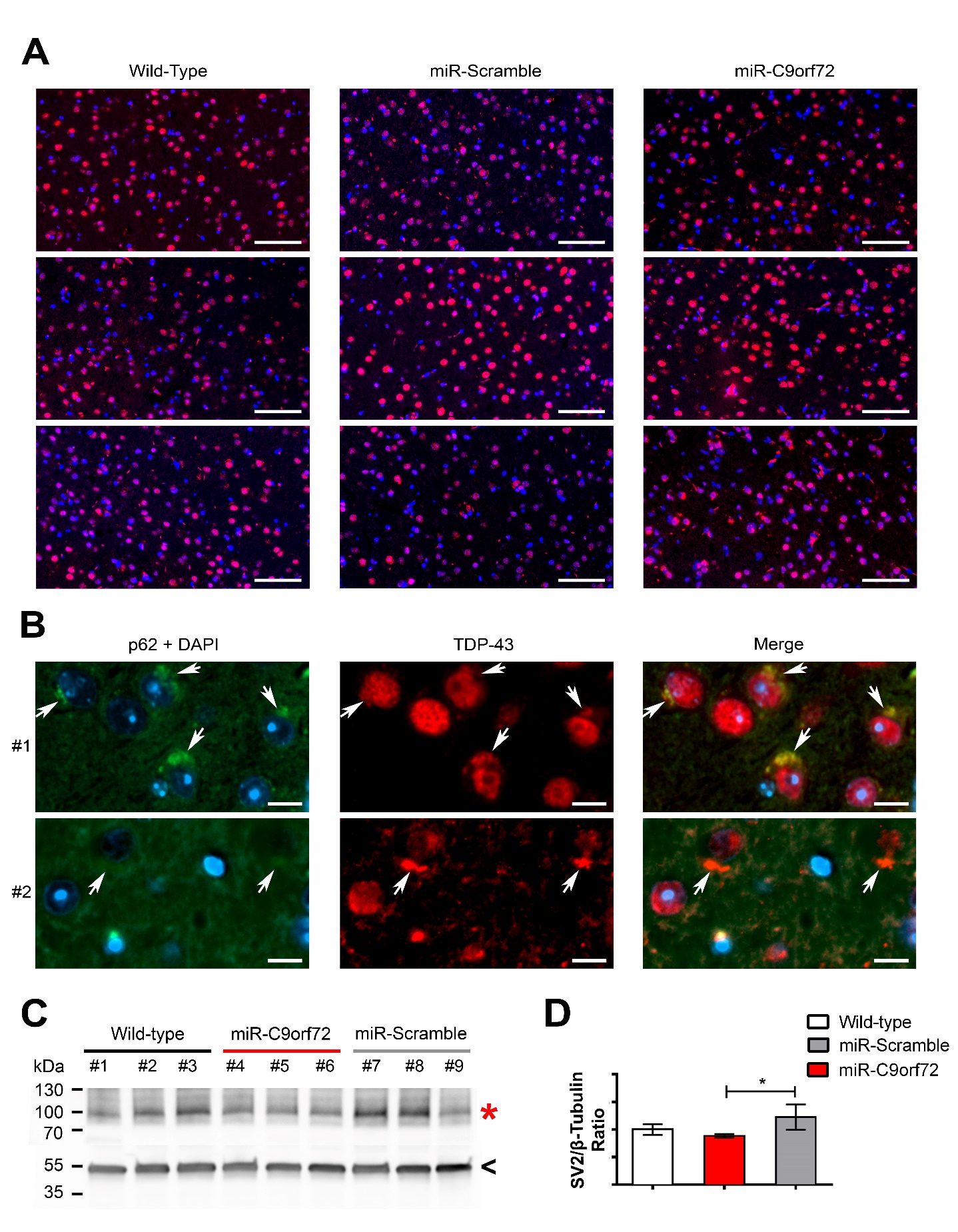


#### Supplementary Figure 2:

1. Representative images of TDP43 staining in 3 animals per genotype in the frontal cortex 2 mm from interaural line. Scale bar: 100µm
2. Representative images of TDP-43 (red) cytoplasmic structures (white arrows) in the frontal cortex from miR-*C9orf72* mice. Some of them colocalize with p62 (green)(see #1), but others do not (see #2). Scale bar: 10µm.
3. Levels of the SV2 protein in the cortex of miR-*C9orf72*, miR-Scramble and wild-type mice.
4. The expression of SV2 (C, red asterisk) was quantified by densitometric analysis of western blots and normalized to β-tubulin (C, black arrowhead). The positions of the molecular weight marker are indicated on the left in kDa.

Error bars represent SEM; *p<0.05


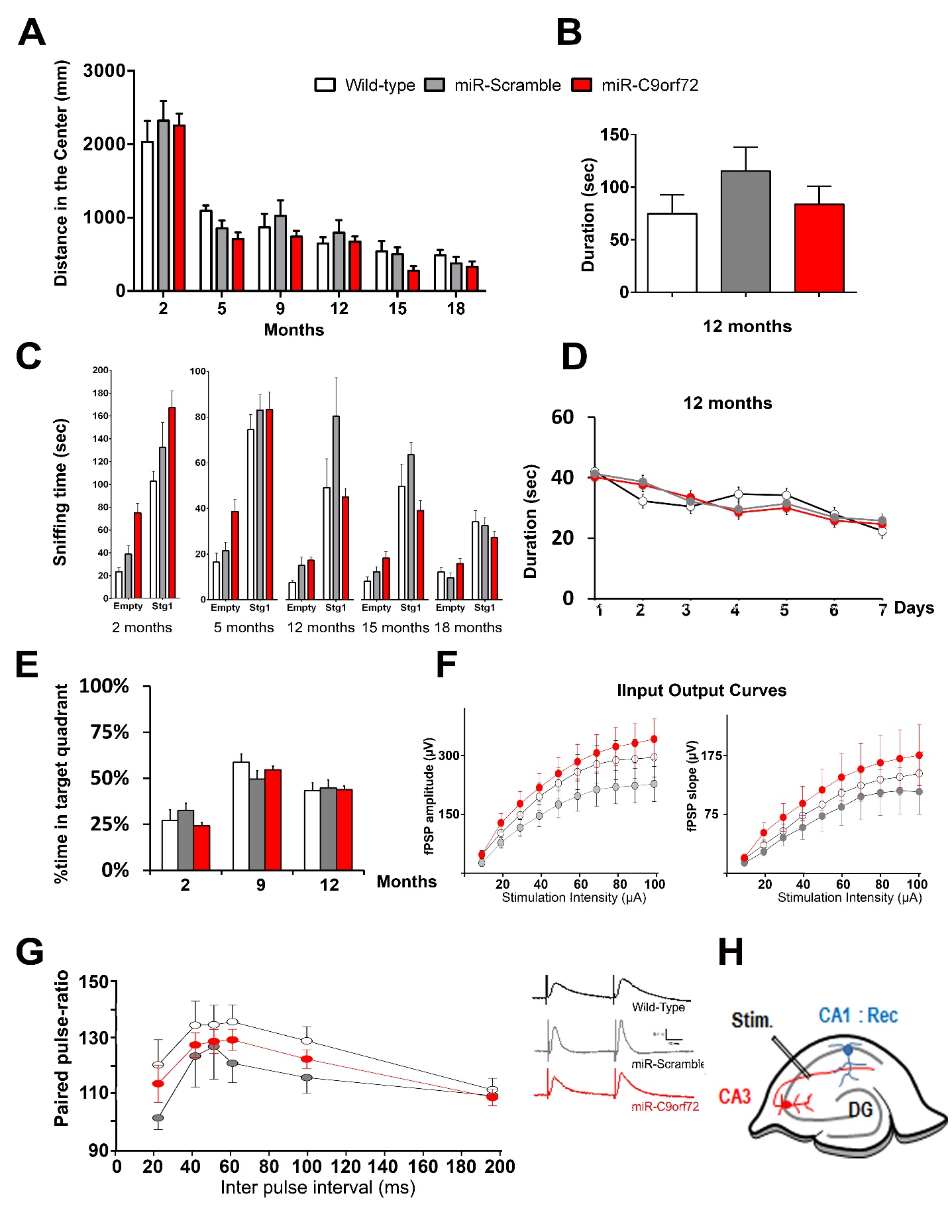


#### Supplementary Figure 3:

1. Open field test at 2-5-9-12-15 and 18 months of age showing activity in the center of the field of C9ORF72 deficient mice and controls (wild-type n=10; miR-Scramble n=12, miR-*C9orf72* n=34)
2. Dark and light test to identify anxiety-like behaviours at 12 months. The time spent in the light chamber is measured (wild-type n=6; miR-Scramble n=6, miR-*C9orf72* n=7).
3. Three-chamber sociability test at 2-5-12 and 18 months measuring social interaction by the mean time spent sniffing an object versus sniffing a mouse. miR-*C9orf72* mice are capable of discerning between an object and a mouse and show normal preference for social interaction.
4. Learning phase in the Morris water maze shows no difference in C9ORF72 deficient mice.
5. Spatial memory testing in the Morris water maze at 2-9 and 15 months shows no memory impairment in miR-*C9orf72* mice.
6. Study of the input-output curve. The CA1 post-synaptic field potential (fPSP) amplitude and slope in response to increasing stimulation of the Shaffer collaterals.
7. Study of the Paired-Pulse Ratio. The CA3-CA1 synapses tend to facilitate when 2 stimulations are delivered with an inter-pulse interval of 40-200ms. No significant difference was detected between the miR-*C9orf72* and the control animals. On the left, representative fPSP traces in response to 2 stimulations delivered at 20Hz in wild-type, miR-*C9orf72* and miR-Scramble animals.
8. Schematic representation of the hippocampic network studied. The CA1 pyramidal neurons were excited by the stimulation of the Shaffer collaterals (red) and the synaptic responses were recorded in the CA1 stratum pyramidale and the stratum radiatum (blue).

Error bars represent SEM.


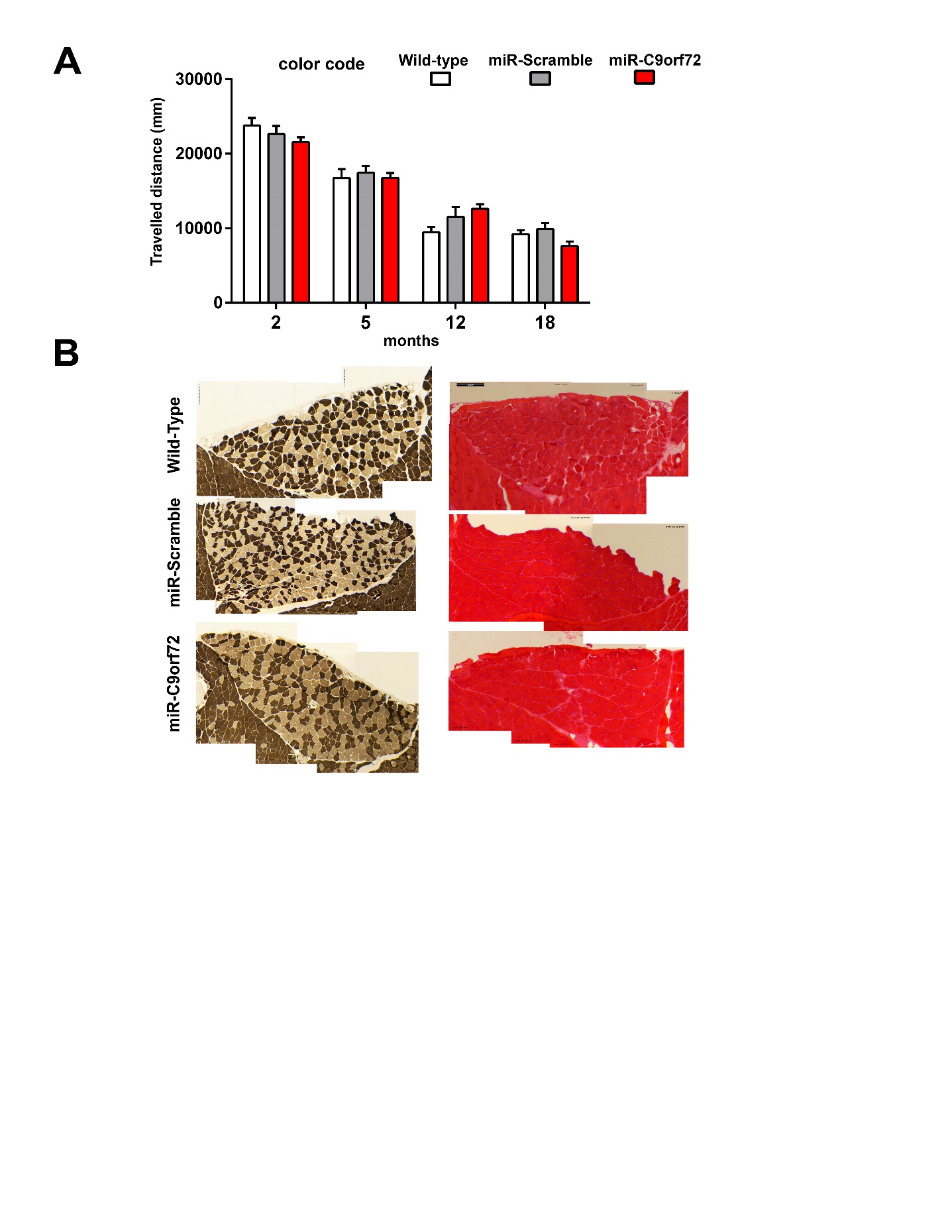


#### Supplementary Figure 4:

1. Open field test at 2-5-12 and 18 months of age showing total activity of C9ORF72 deficient mice and controls (wild-type n=10; miR-Scramble n=12, miR-*C9orf72* n=34)
2. Muscle characterization by ATPase staining at pH 9,4 (left) and H&E staining (right) at in 23 months old mice show not difference (n=4 per group)

Error bars represent SEM.
